# Refinement of the MHC Risk Map in a Scandinavian Primary Sclerosing Cholangitis Population

**DOI:** 10.1371/journal.pone.0114486

**Published:** 2014-12-18

**Authors:** Sigrid Næss, Benedicte A. Lie, Espen Melum, Marita Olsson, Johannes R. Hov, Peter J. P. Croucher, Jochen Hampe, Erik Thorsby, Annika Bergquist, James A. Traherne, Erik Schrumpf, Kirsten Muri Boberg, Stefan Schreiber, Andre Franke, Tom H. Karlsen

**Affiliations:** 1 Norwegian PSC Research Center, Department of Transplantation Medicine, Division of Cancer Medicine, Surgery and Transplantation, Oslo University Hospital, Rikshospitalet, Oslo, Norway; 2 Institute of Clinical Medicine, University of Oslo, Oslo, Norway; 3 K.G Jebsen Inflammation Research Centre, Research Institute of Internal Medicine, Oslo University Hospital, Rikshospitalet, Oslo, Norway; 4 Department of Immunology, Oslo University Hospital, Rikshospitalet, Oslo, Norway; 5 Department of Medical Genetics, University of Oslo and Oslo University Hospital, Oslo, Norway; 6 Mathematical Sciences, Chalmers University of Technology, Gothenburg, Sweden; 7 Department of Environmental Science, Policy, and Management, University of California Berkeley, Berkeley, California, United States of America; 8 First Medical Department, University Hospital Dresden, Technical University Dresden, Dresden, Germany; 9 Department of Gastroenterology and Hepatology, Karolinska Institutet, Karolinska University Hospital, Huddinge, Stockholm, Sweden; 10 Division of Immunology, Department of Pathology, University of Cambridge, Cambridge, United Kingdom; 11 Cambridge Institute for Medical Research, University of Cambridge, Cambridge, United Kingdom; 12 Section for Gastroenterology, Department of Transplantation Medicine, Division of Cancer Medicine, Surgery and Transplantation, Oslo University Hospital, Rikshospitalet, Oslo, Norway; 13 Institute of Clinical Molecular Biology, Christian-Albrechts-University, Kiel, Germany; 14 Department of General Internal Medicine, Christian-Albrechts-University, Kiel, Germany; Hospital Israelita Albert Einstein, Brazil

## Abstract

**Background:**

Genetic variants within the major histocompatibility complex (MHC) represent the strongest genetic susceptibility factors for primary sclerosing cholangitis (PSC). Identifying the causal variants within this genetic complex represents a major challenge due to strong linkage disequilibrium and an overall high physical density of candidate variants. We aimed to refine the MHC association in a geographically restricted PSC patient panel.

**Methodology/Principal Findings:**

A total of 365 PSC cases and 368 healthy controls of Scandinavian ancestry were included in the study. We incorporated data from HLA typing (*HLA-A, -B, -C, -DRB3, -DRB1, -DQB1*) and single nucleotide polymorphisms across the MHC (n = 18,644; genotyped and imputed) alongside previously suggested PSC risk determinants in the MHC, i.e. amino acid variation of DRβ, a *MICA* microsatellite polymorphism and *HLA-C* and *HLA-B* according to their ligand properties for killer immunoglobulin-like receptors. Breakdowns of the association signal by unconditional and conditional logistic regression analyses demarcated multiple PSC associated MHC haplotypes, and for eight of these classical HLA class I and II alleles represented the strongest association. A novel independent risk locus was detected near *NOTCH4* in the HLA class III region, tagged by rs116212904 (odds ratio [95% confidence interval] = 2.32 [1.80, 3.00], *P* = 1.35×10^−11^).

**Conclusions/Significance:**

Our study shows that classical HLA class I and II alleles, predominantly at *HLA-B* and *HLA-DRB1*, are the main risk factors for PSC in the MHC. In addition, the present assessments demonstrated for the first time an association near *NOTCH4* in the HLA class III region.

## Introduction

Primary sclerosing cholangitis (PSC) is a rare (prevalence 1/10,000) liver disease resulting in chronic inflammation and concentric fibrosis of the intra- and extra hepatic bile ducts. Medical therapy does not halt disease progression and up to 50% of the patients are in need of liver transplantation within 10–15 years of diagnosis [1]. PSC is often associated with other immune-mediated diseases; most frequently with inflammatory bowel disease (IBD). Co-occurrence of IBD shows geographical variation, with highest frequencies of IBD occurring in Scandinavia (approximately 80%) and lower frequencies in Southern Europe and Asia (approximately 30–50%). Other immune-mediated comorbidities such as type 1 diabetes, autoimmune thyroid disorders, psoriasis and rheumatoid arthritis are found in up to 25% of the patients [2] and shows a partially overlapping genetic predisposition [3].

The presence of genetic components in PSC susceptibility is suggested by an increased disease risk in siblings of PSC patients (9–39 fold). The statistically most significant genetic associations are located within the major histocompatibility complex (MHC) on chromosome 6p21. This genetic complex spans almost 4 million base pairs (Mbp), harboring approximately 260 genes. Many of these genes have immune-related functions, the most important being the classical HLA class I and II genes [4]. A strong genetic association within the MHC, as observed in PSC, is a hallmark of most autoimmune and immune-mediated diseases. Due to the complexity of the region, including extensive linkage disequilibrium (LD), population heterogeneity and the high density of immune-related genes, it has for most diseases been difficult to pinpoint the causal genetic variant(s) and hence the functional implications of the genetic associations. Exceptions exist for which a primary MHC risk factor has been identified, including HLA-DQ2 and -DQ8 in celiac disease [5] and HLA-DQ*06:02 in narcolepsy [6]. Recent advances in single nucleotide polymorphism (SNP) genotyping technologies with dense coverage in the MHC and *in silico* imputation of missing SNP genotypes [7] as well as classical HLA alleles and their corresponding amino acid variants [8], [9], have facilitated refinement of MHC associations in several diseases. This has proven most successful for diseases with one prominent predisposing genetic factor mapping to either the class I or II region, as observed in type 1 diabetes [10], [11], rheumatoid arthritis [12] and multiple sclerosis [13]. For other MHC associated diseases, such as PSC, the challenges associated with refinement of the association signal are substantial, mainly due to the lack of a primarily associated locus, and the presence of multiple conserved haplotypes [14]–[17].

Against this background, we aimed to refine the MHC associations in a Scandinavian PSC population by exploring the extent of the various associated haplotypes and to determine whether additional associations with non-classical HLA genes could be detected. Two different approaches were applied. As no primary HLA predisposition is known in PSC, we first set out to identify a putative main susceptibility locus by unconditional multivariate logistic regressions of data for six classical HLA class I and II genes (*HLA*-*A, HLA-B, HLA-C, DRB3*, *DRB1* and *DQB1*) obtained by direct sequencing. Next, HLA sequencing data, more than 18,000 genotyped and imputed SNPs within the MHC and information on previously reported associations with amino acid variation at position 37 and 86 of the *DRB1* locus [18], the MICA5.1 allele [19], [20] and *HLA-B* and *HLA-C* killer immunoglobulin-like receptors (KIR) ligands (Bw4/Bw6 and C1/C2, respectively) [19], were jointly considered in stepwise conditional logistic regression models.

## Materials and Methods

### Study population

The study included 365 Scandinavian PSC patients recruited on admission to Department of Transplantation Medicine, Oslo University Hospital, Rikshospitalet, Oslo, Norway (n = 232) and Department of Gastroenterology and Hepatology, Karolinska University Hospital, Huddinge, Stockholm, Sweden (n = 133). The diagnosis of PSC was based on generally accepted clinical, biochemical, cholangiographic and histological criteria [1]. IBD was diagnosed and classified according to standard endoscopic and histologic criteria and was present in 86% of the patient population. A diagnosis of cholangiocarcinoma had been made in 14% of the patients. Median age at diagnosis of PSC was 34 years (range 12–75). An ethnically and gender-matched group (70% males) of healthy controls (n = 368) was randomly drawn from the Norwegian Bone Marrow Donor Registry and anonymized after DNA retrieval. The registry comprises more than 25,000 individuals with a median age (range) of 38 (18–55) years. Our study was restricted to the Scandinavian population to minimize the effect of population ancestry and the variable degree of non-PSC immune-mediated co-morbidity on MHC haplotype architecture.

Written informed consent was obtained from all study participants. The study was approved by The Regional Committee for Research Ethics in Southern Norway and The Ethics Committee of Karolinska Institutet, Stockholm, Sweden.

### HLA genotyping

For HLA class II, sequencing based *HLA-DRB3* typing was performed using previously described protocols [21], utilizing AssignSBT v3.2.7b for allele assignment [22]. Four digit genotyping results were available for *HLA-DRB1* and *HLA-DQB1* from a previous study that included the same patients and controls [23]. For HLA class I, *HLA-A, HLA-B* and *HLA-C* genotypes were available from another previous study that included the same patients and controls [19]. For *HLA-A* and *HLA-B*, alleles were collated at serotype nomenclature level to reduce the number of degrees of freedom in the logistic regression analysis and to ensure comparability with the control data from the Norwegian Bone Marrow Donor registry. Data on the MICA 5.1 variant as well as HLA-B and HLA-C KIR binding epitopes (HLA-Bw4 and HLA-Bw6, HLA-C1 and HLA-C2, respectively) were also available from other previous studies [19], [20].

### MHC SNP genotyping and 1000G imputation

The SNPlex Genotyping System (Applied Biosystems) was used to genotype 471 SNPs spanning 16 MB (27.7–44.1 Mbp) covering the MHC (29,6–33,1 Mbp) [24]. The SNPs were selected based on published allele frequencies and genomic location. Genotyping cluster-plots were manually inspected and poor performing SNPs were excluded prior to further analyses. SNPs with low genotyping success rate (<95%), minor allele frequency (MAF) <1% and deviating from Hardy-Weinberg equilibrium in controls (*P*-value <1×10^−4^), were excluded. A total of 405 SNPs passed quality control and served as input for imputation. Replication genotyping of rs116212904 was performed using TaqMan technology (Applied Biosystems).

For SNP imputation, we used the 1000 genomes reference data from individuals of European ancestry (1000G Phase I v3 20101123, http://www.sph.umich.edu/csg/abecasis/MACH/download/1000G.2012-03-14.html). This dataset consists of 758 phased haplotypes. Mach 1.0 was used to determine haplotypes based on the genotyped SNPs [25], followed by genotype imputation using minmac v. 2012.5.29 [26], [27]. For downstream analyses only imputed SNPs with a MAF >1% and *r* ^2^ values above a MAF-specific *r* ^2^ threshold were employed. To determine the MAF-specific *r* ^2^ threshold, the SNPs were split into three MAF groups (1)>1% and <3%, 2)>3% and <5% and 3)> 5%). The *r* ^2^ threshold in the respective groups was set so the average *r* ^2^ was above 0.8. After applying these criteria 18,644 SNPs (27.6–44.2 Mbp) were included in the statistical analyses.

The imputed dataset was converted to PLINK format using fcgene v.1.0.5, subsequent LD analyses were done in Unphased v.3.0.10 and PLINK v.1.07 using the imputed best-guess genotypes.

### Statistical analyses

#### Unconditional regressions including classical HLA class I and II alleles

One hypothesis is that the multiple associations previously reported in PSC are caused by LD to primary associations to alleles at a single classical HLA locus. A stepwise logistic regression approach [28] was used to test if allelic variation at one of the six HLA loci; *HLA-A*, *HLA-B*, *HLA-C*, *HLA-DRB3, HLA-DRB1* or *HLA-DQB1*, could fully explain the HLA associations observed in PSC. The contribution to the model of each of the loci were tested using a likelihood ratio test, one at the time, comparing with a model containing each of the six loci as baseline.

In PSC, no primary risk locus has been established. This complicates the discrimination between potentially multiple, independent risk alleles in the MHC. To determine which of the alleles, across *HLA-A*, *HLA-B*, *HLA-C*, *HLA-DRB3, HLA-DRB1* and *HLA-DQB1*, could best predict case-control status, the stepAIC function in R (http://cran.r-project.org/web/packages/MASS/index.html) was applied using all alleles at all six loci simultaneously. Due to strong LD between *DRB1* and *DQB1* alleles in the Scandinavian population, these were combined and assessed as *DRB1-DQB1* haplotypes to reduce the degrees of freedom. The method selects, at each step the covariate that, by exclusion or inclusion to the regression model, decreases the Akaike Information Criterion (AIC) the most. To avoid overfitting, we only kept the covariates that gave a significant contribution using the likelihood ratio test and nominal significance threshold of *P*≤0.01.

#### Conditional logistic regressions

Next, we wanted to comprehensively assess previously reported PSC associations in the MHC together with classical HLA alleles and SNP data spanning the MHC, in one model. To determine the relative impact of significant outcomes, stepwise conditional logistic regressions were performed. Analyses were done in R v.3.0.1 with custom scripts (available from the authors on request). In total, 18,771 variables, consisting of the 18,644 SNPs, 95 HLA alleles (at *HLA-A*, *HLA-B*, *HLA-C*, *HLA-DRB3, HLA-DRB1* and *HLA-DQB1*), 20 *DRB1-DQB1* haplotypes, amino acid polymorphisms at position 37 (five) and 86 (two) of *DRB1*, the MICA5.1 allele and the four KIR epitopes, were individually tested for association in logistic regression with a genetic additive model. To account for the uncertainty in imputation, allele dosages from the imputation procedure represented the different SNP genotypes. HLA alleles, *DRB1-DQB1* haplotypes, amino acid variants, the MICA5.1 allele and the KIR epitopes were converted into dosages for each individual, i.e. 0, 1, or 2 copies. Amino acids at position 37 (Asparagine, Leucine, Phenylalanine, Serine, Tyrosine) and 86 (Glycine, Valine) were assigned by aligning *DRB1* alleles in the IMGT/HLA database release 3.11.0. A study-specific Bonferroni-corrected statistical significance threshold of *P*≤2.66×10^-6^ was set, according to the number of comparisons.

## Results

### The MHC association in PSC can only be explained by multiple independent variants

Many classical HLA genes and alleles have previously been reported to be associated with PSC [29]. In an attempt to dissect a potential primary effect within the MHC, either for classical HLA-loci or non-classical HLA loci, we applied (i) unconditional multivariate regression models and (ii) conditional univariate regressions.

### Unconditional multivariate regressions result in independent HLA class I and II associations

In the first strategy, we started by assessing whether the HLA associations in PSC could be restricted to alleles at only one of the six classical HLA-loci genotyped (i.e. *HLA-A, HLA-B, HLA-C, DRB3, DRB1* and *DQB1*). This was not the case since alleles at *HLA-B, DRB1, DRB3* and *DQB1* all provided statistically significant contributions to the model over and above any of the other loci alone.

Secondly, we investigated which alleles at *HLA-A, HLA-B, HLA-C* and *DRB3* (n = 46), and which *DRB1-DQB1* haplotypes (n = 20), could represent primary disease determinants by incorporating these 66 variants in regression modeling. Several HLA alleles at different loci were significantly associated in the final model (i.e. *P*≤0.01) (Table 1). At HLA class I, HLA-B*08 (i.e. serotype B8) (OR [95% CI] = 4.3 [3.0, 6.0]), -B*07 (i.e. serotype B7) (OR [95% CI] = 1.7 [1.2, 2.4]) and -C*06 (OR [95% CI] = 2.5 [1.4, 4.5]) all conferred risk of PSC. B*08 and B*07 are both representatives of conserved MHC haplotypes; i.e. the ancestral haplotype 8.1(AH8.1) and the ancestral haplotype 7.1 (AH7.1), respectively. Notably, the *DRB1* alleles found on these haplotypes, DRB1*03:01 (AH8.1) and DRB1*15:01 (AH7.1), did not show significant contributions over and above that exerted by the HLA-B*08 and HLA-B*07 alleles, respectively. At HLA class II, there were significant associations with both protective and risk *DRB1-DQB1* haplotypes. The DRB1*13:01–DQB1*06:03 (OR [95% CI) = 4.9 [3.1, 7.9]) association was the strongest of the class II haplotypes, and the only class II haplotype conferring risk in the final model. Three class II *DRB1*-*DQB1* haplotypes; DRB1*04–DQB1*03 (OR [95% CI] = 0.4 [0.3, 0.6]), DRB1*07:01–DQB1*03:03 (OR [95% CI] = 0.1 [0.02, 0.6]) and 13:XX (i.e. all non-13:01 alleles)–DQB1*06 (OR [95% CI] = 0.3 [0.3, 0.6]), had a protective effect.

**Table 1:** Unconditional, multivariate logistic regression modeling incorporating all detected alleles from sequencing-based classical HLA typing at six loci.

HLA alleles in final model	OR (95% CI)
*HLA class I loci:*	
HLA-B*08	4.3 (3.0–6.0)
HLA-B*07	1.7 (1.2–2.4)
HLA-C*06	2.5 (1.4–4.5)
*DRB1-DQB1 blocks:*	
DRB1*13:01-DQB1*06:03	4.9 (3.1–7.9)
DRB1*04-DQB1*03	0.4 (0.3–0.6)
DRB1*07:01-DQB1*03:03	0.1 (0.02–0.6)
DRB1*13:XX-DQB1*06^a^	0.3 (0.3–0.6)

Significant contributions to disease status were established on the basis of a likelihood ratio test of the impact from each allele on the Akaike Information Criterion (*P*≤0.01). Due to the strong linkage disequilibrium (LD) between *DRB1* and *DQB1* alleles, and to reduce the degree of freedom of the test, they were modeled as “*DRB1-DQB1* blocks” according to LD. ^a^DRB1*13:XX  =  all non-13:01 alleles, i.e. DRB1*13:02, 13:03 and 13:05.

CI: confidence interval, OR: odds ratio

In summary, this first strategy was not able to identify one primary classical HLA effect, but rather indicated that several HLA class I and II alleles are independently associated with PSC. Furthermore, the analysis demonstrated the presence of true protective effects as well as susceptibility effects, i.e. that the DRB1*04, DRB1*07 and DRB1*13:02/DRB1*13:03 associations do not arise because of the high frequencies of the risk alleles, but are independent from these (Table 1 and S1 Table).

### Conditional analyses across the MHC confirm independent associations with HLA class I and II alleles and detected a novel independent association to an allele in the HLA class III region

In the second analytic strategy, we assessed the relative importance of several PSC-associated polymorphisms with various biological implications, including SNPs, alleles encoding classical HLA molecules and non-classical HLA molecules, ligands for receptors located on natural killer cells and key amino acid variants possibly influencing peptide presentation of the *DRB1* molecule.

**Figure 1:**
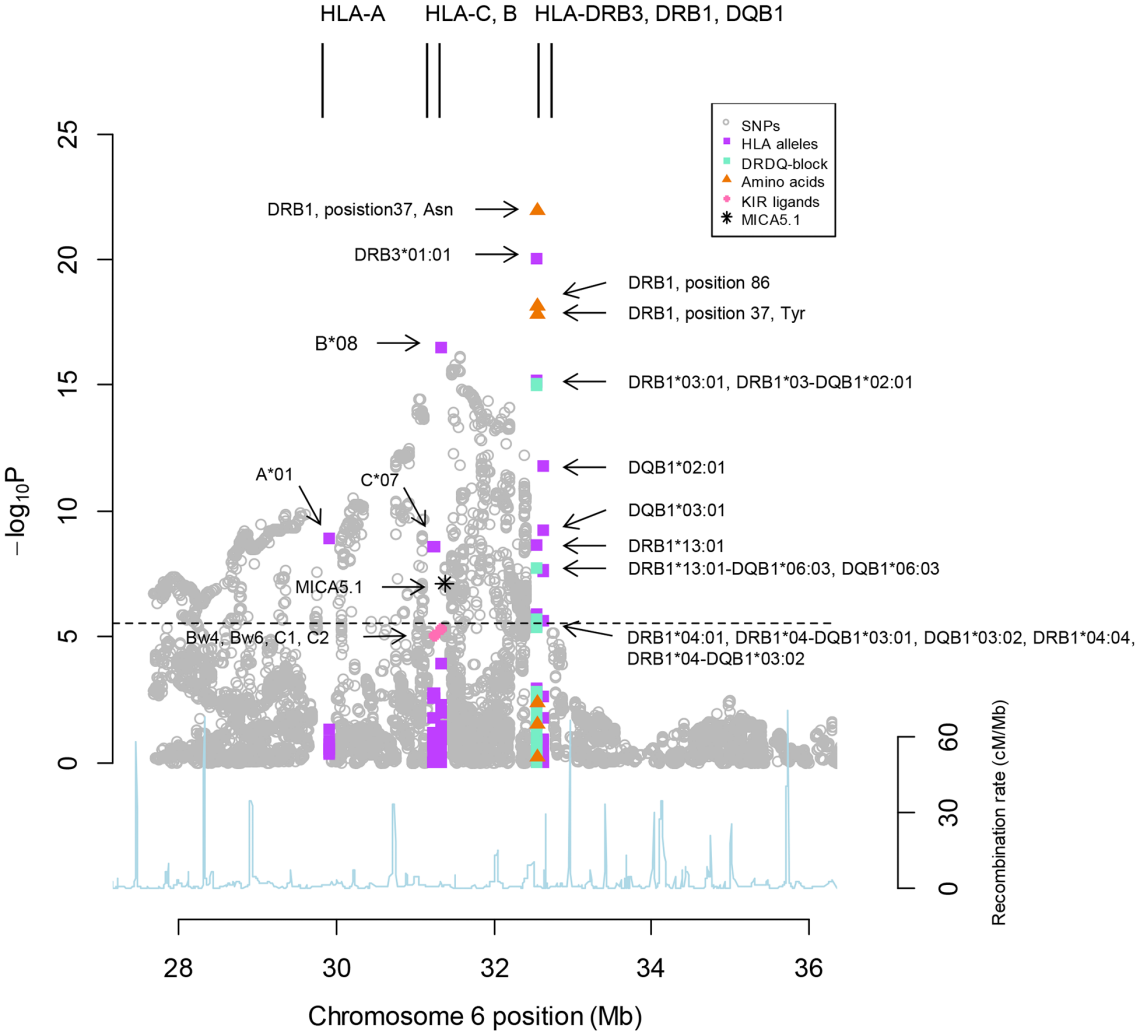
Single marker association results for presently and previously implicated major histocompatibility complex (MHC) risk variants in primary sclerosing cholangitis (PSC). The strength of association signal, -log_10_P-value (y-axis), is plotted against the position on chromosome 6 shown in million base pairs (Mb, x-axis) for each of the 18,771 variables. The black, horizontal dotted line represents the Bonferroni-corrected statistical significance threshold of *P*≤2.66×10^−6^. Association results are categorized according to single nucleotide polymorphisms (SNPs), classical HLA alleles, *DRB1-DQB1* haplotypes, previously reported amino acids, MICA5.1, as well as variants affecting HLA-B (i.e. Bw4 and Bw6) and HLA-C (i.e. C1 and C2) ligand abilities for killer immunoglobulin-like receptors (KIR). The blue line shows recombination intensity (cM/Mb). Positions are given according to National Center for Biotechnology Information's build 37 (hg19).

Fig. 1 shows the primary association results within the MHC, including all tested variants. The statistically most significant association was found for DRβ amino acid position 37, i.e. Asparagine (OR [95% CI] = 3.30 [2.62, 4.17], *P* = 1.11×10^−22^). The most significant association found for a HLA allele was with DRB3*01:01 (OR [95% CI] = 3.51 [2.73, 4.51], *P* = 9.45×10^−21^), and the most significant SNP association was with rs139345387 at 31.6 Mb (OR [95% CI] = 0.37 [0.29, 0.48], *P* = 6.85×10^−17^). Several *HLA-DRB1* alleles in our population encode Asparagine at position 37, including HLA-DRB1*03:01, *09:01, *13:01, *13:02 and *14:02. Two of these alleles, DRB1*13:01 (*P* = 2.30×10^−9^), and *03:01 (*P* = 6.82×10^−16^), were significantly associated in the primary association analysis (Fig. 1). The most strongly associated HLA-allele, HLA-DRB3*01:01, was found on two PSC risk haplotypes; AH8.1 (i.e. A*01-B*08-C*07-DRB3*01:01-DRB1*03:01-DQA1*05:01-DQB1*02:01) and DRB1*13:01-DQB1*06:03 in our study population. DRB3*01:01 is however only in substantial LD with alleles on the AH8.1 (*r* ^2^ = 0.69 with DRB1*03:01 in controls) and not DRB1*13:01. This means that *DRB3* alleles other than DRB3*01:01 are observed together with DRB1*13:01, and conversely, as evident from the AIC-based regressions in the first analytic strategy, that DRB1*13:01 provides an effect over and above that exerted by DRB3*01:01. The top 10 SNPs in the primary association analysis (including rs139345387) were all part of the same LD-block and in considerable LD with the AH8.1 alleles, most strongly with HLA-B*08 (*r* ^2^ ranging from 0.43 to 0.83). Our peak SNP signal aligns with previous studies where the top SNP associations were found in/or in the vicinity of *HLA-B*, tagging HLA-B*08 [3], [30], [31]. Given the different biological implications of the various polymorphisms included in this second strategy, one can argue in favor of various subsequent conditional strategies in a follow-up of the primary association analysis. Here we present three different strategies.

#### Conditional analysis strategy I

**Figure 2:**
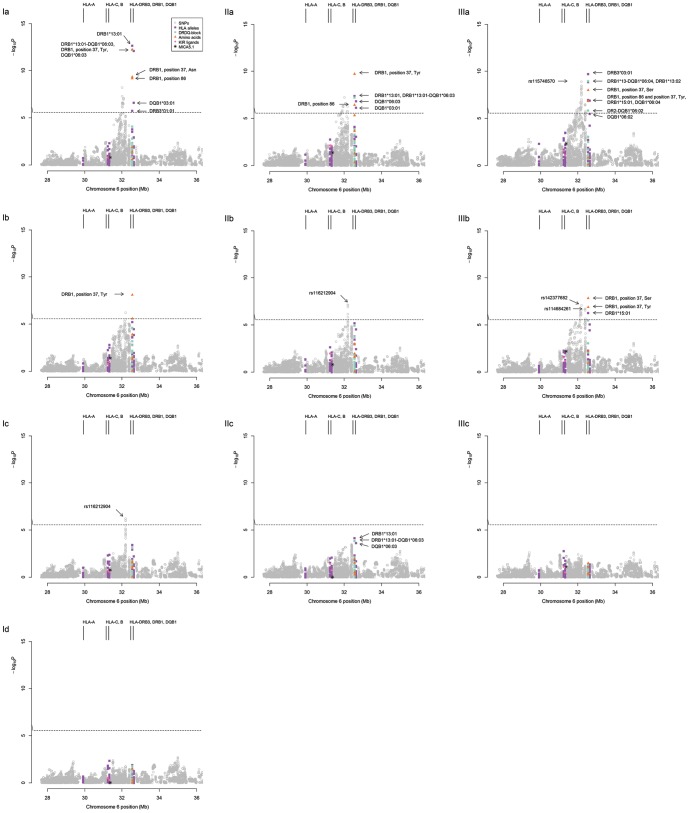
Conditional logistic regression analyses within the major histocompatibility complex (MHC) in primary sclerosing cholangitis (PSC). The three various conditional strategies employed and their conditional steps are indicated by Ia-Id, IIa-IIc and IIIa-IIIc. For all panels the conditional *P*-values (y-axis) are plotted against the position on chromosome 6 shown in million base pairs (Mb, x-axis). HLA alleles, *DRB1-DQB1* haplotypes, SNPs, the MICA5.1 allele and KIR ligands are distinctly designated (see legend in panel Ia). The significance threshold (*P*≤2.66×10^−6^) is indicated by the black, horizontal dotted line.

**Figure 3:**
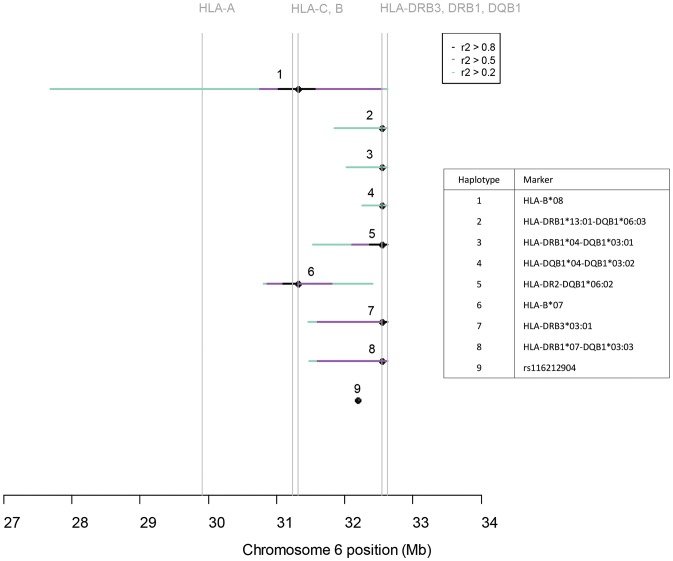
The extension of nine significantly primary sclerosing cholangitis (PSC) associated major histocompatibility complex (MHC) haplotypes, observed in the unconditional and conditional regression analyses combined. The extension of the haplotypes is determined by three categories of linkage disequilibrium (*r* ^2^>0.2 (green), *r* ^2^>0.5 (purple) and *r* ^2^>0.8 (black), respectively) between a single haplotype marker (listed in the figure box) and any single nucleotide polymorphism (SNP) in the dataset. Haplotype 1; AH8.1 (A*01-C*07-B*08-DRB3*01:01-DRB1*03:01-DQA1*05:01-DQB1*02:01). Haplotype 2; HLA-DRB1*13:01-DQB1*06:03. Haplotype 3; HLA-DRB1*04-DQB1*03:01. Haplotype 4; HLA-DRB1*04-DQB1*03:02. Haplotype 5; HLA-DRB1*15:01-DQB1*06:02. Haplotype 6; HLA-B*07. Haplotype 7; HLA-DRB3*03:01-DRB1*13:02-DQB1*06:04. Haplotype 8, HLA-DRB1*07:01-DQB1*03:03. Haplotype 9; an independent LD-block centromeric of *NOTCH4* in class III, tagged by rs116212904. DRB1*15:01 is part of the DR2 serotype group.

We started with the AH8.1, and more specifically HLA-B*08, as the first conditional variable. This allele was tagged through LD by the most significantly associated SNP allele, and was also represented in the final model of the classical HLA alleles assessed by global AIC (Table 1). After adjusting for the HLA-B*08 association, several statistically significant association signals remained in the analysis (Fig. 2.Ia). The top signal was with components of the DRB1*13:01-DQB1*06:03 haplotype, represented by the DRB1*13:01 allele (*P* = 2.25×10^−13^). Adjusting for both the HLA-B*08 and HLA-DRB1*13:01 association resulted in an association with Tyrosine at position 37 (Tyr37) of the DRβ molecule (*P* = 7.99×10^−9^) (Fig. 2.Ib). Tyr37 is in LD with the DRB1*04 and DRB1*11 alleles, both of which show reduced frequencies in PSC patients (S1 Table and S2d Table in S2 Table). Non-associated alleles also exhibit Tyr37, notably, DRB1*08, DRB1*10:01 and DRB1*13:03. Adjusting for the B*08, DRB1*13:01 and Tyr37 associations in the analysis resulted in a final, distinct residual signal in the class III region (Fig. 2.Ic). The top SNP, rs116212904, achieved a significance level of *P* = 1.35×10^−11^ in the primary association analysis, and *P* = 5.94×10^−7^ in this final step of the first conditional analysis. This biallelic A/T SNP was one of the 405 genotyped SNPs that served as input for imputation. To validate the association, we genotyped the same study population employing TaqMan technology and obtained comparable frequencies of the A allele of 81% and 66% in PSC and controls respectively (OR [95% CI) = 2.28 [1.79–2.91], *P* = 1.67×10^−11^). rs116212904 (renamed to rs9267845) is part of a LD-block in the class III region, located centromeric of the *NOTCH4* gene. Notably, weak LD was observed between this block and any of the PSC-associated HLA alleles, *DRB1-DQB1* haplotypes or amino acids tested in this study (illustrated in Fig. 2.Ic and Fig. 3). This observation was supported by persistent association of all the major risk and protective variants when correcting for the rs116212904 association (S1 Fig.). Concluding strategy I, when conditioning on all four variables (i.e. B*08, DRB1*13:01, Tyr37 and rs116212904) in the regression analysis, no residual associations were left within the MHC (Fig. 2.Id).

#### Conditional analysis strategy II

A second conditional approach was performed by selecting the most significant HLA-allele; DRB3*01:01, as the primary conditioning variable, resulting in a top peak at Tyr37. Since DRB3*01:01 is part of the AH8.1, the observed elimination of the B*08 association (P-value dropping from 3.14×10^−17^ to 0.02) was expected (Fig. 2.IIa). Although DRB3*01:01 is observed on some DRB1*13:01-DQB1*06:03 haplotypes, we would not expect this association signal to drop accordingly, due to lack of LD. A marginal drop in *P*-value for DRB1*13:01 from 2.30×10^−9^ to 4.03×10^−8^ confirmed this prediction, indicating an independent association of DRB1*13:01-DQB1*06:03, irrespective of DRB3*01:01 status. Further conditioning on both DRB3*01:01 and Tyr37 resulted in a significant residual association signal within class III, peaking at rs116212904 (Fig. 2.IIb), the same SNP identified in the first conditional strategy. Conditioning on DRB3*01:01, Tyr37 and rs116212904 left no significant residual association signals within the MHC (Fig. 2.IIc). However, a non-significant association (according to the study-specific significance threshold) was observed with DRB1*13:01-DQB1*06:03 (*P* = 7.11×10^−5^ for DRB1*13:01), indicating the presence of residual effects represented by this haplotype, and not corrected for in this second conditional strategy.

#### Conditional analysis strategy III

In the third and final conditional approach, the most significant association in the primary association analysis, Asparagine at position 37 (Asn37) of *DRB1*, was selected as the first conditional variable. This resulted in significant residual associations for one protective, i.e. DRB3*03:01-DRB1*13:02-DQB1*06:04, and one risk haplotype, DRB1*15:01-DQB1*06:02 (Fig. 2.IIIa). In addition there was a significant residual signal for Serine at position 37 of *DRB1* (Ser37), an amino acid encoded by several DRB1*01 alleles (01:01, 01:02, 01:03 and 01:07), in addition to DRB1*15:02, DRB1*16:01 and the previously and presently implicated DRB1*15:01 allele [16]. We detected the same distinct peak as observed in strategy I in the class III region, represented by rs115746570 (*P* = 1.23×10^−9^). This SNP was the ninth most significant SNP in the third step of the first conditional strategy (Fig. 2.Ic) and LD implies that it represents the same association located centromeric to *NOTCH4* as detected in the first two conditional approaches. After conditioning on Asn37 and DRB3*03:01, significant residual associations were detected for Serine and Tyrosine at position 37, DRB1*15:01 and two further SNPs (one centromeric of *NOTCH4* and another located between *BTNL2* and *HLADRA*) (Fig. 2.IIIb). Conditioning on Asn37, HLA-DRB3*03:01 and Ser37, resulted in no significant residual PSC association signal within the MHC (Fig. 2.IIIc).

In summary, this second analytic strategy resulted in six associated HLA haplotypes, three risk; i.e. AH8.1 (A*01-B*08-C*07-DRB3*01:01-DRB1*03:01-DQA1*05:01-DQB1*02:01), DRB1*13:01-DQB1*06:03 and DRB1*15:01-DQB1*06:02, and three protective; i.e. DRB1*04-DQB1*03:01, DRB1*04-DQB1*03:02 and DRB3*03:01-DRB1*13:02-DQB1*06:04. In addition, a significant and independent association was found in class III in the vicinity of *NOTCH4* tagged by rs116212904.

Fig. 3 shows the extension, as determined by LD with any SNP in the dataset, of nine significantly associated haplotypes from the first and second analytic strategy combined.

## Discussion

By combining novel unconditional multivariate logistic regression analyses and three conventional conditional logistic regression strategies, we were able to carefully characterize the presence and relationship of independent class I, class II and class III contributions to the MHC associations in a Scandinavian PSC population at an unprecedented level. A previously unreported class III association centromeric to *NOTCH4* was evident regardless of the statistical approach taken to assess the data.

A new finding in our study is the independent association located in the class III region. The top seven SNPs (*P*-value ≤2.66×10^−6^) span a 1634 bp long region (32193220 to 32194854), incorporating a non-coding region centromeric of *NOTCH4*. There are several possible functional interpretations of this association. The notch signaling pathway has numerous biological functions during the development of the central nervous system and vascular system, and also appears to play a role in the differentiation of naïve CD4+ T cells [32]. Several diseases have reported associations either within or in the vicinity of *NOTCH4*, most extensively schizophrenia [33]. The second most significant SNP (rs115991177) in conditional strategy one and two has previously been found to be associated with type 1 diabetes, [34] and is in perfect LD (*r* ^2^ = 1) with our top SNP (rs116212904). rs116212904 is located 1376 base pairs centromeric of *NOTCH4*. However, SNPs from the 1000G EUR population in moderate LD (i.e. *r* ^2^≥0.2) with rs116212904 span a large region (∼400 kb), from 32191457 to 32594039, and encompasses several class III and class II genes. Hence, the identified class III association could potentially tag causal variants in genes other than *NOTCH4*, including *HLA-DRB1*. Yet, tagging of classical HLA alleles seems unlikely, as there was only weak LD between rs116212904 and any of the sequenced HLA class I or II alleles in our control population. Furthermore, selecting rs116212904 as the first conditional variable left significant residual signals for all the associated HLA-alleles, *DRB1*-*DQB1* haplotypes and amino acid variants found in the primary association analysis. Finally, rs116212904 was also highly significant with a *P*-value of 1.62×10^−59^ in the previously published PSC Immunochip study [3], although fine-mapping efforts were not undertaken in that study.

The association results for classical HLA class I and II alleles and haplotypes from the unconditional and conditional logistic regressions are consistent with previous work in the MHC in PSC, yet significantly extend our knowledge regarding several of the risk haplotypes. Importantly, the HLA-B*08 and -B*07 alleles that remained in the final model of the unconditional regressions are part of haplotypes which are highly conserved in the general population, stretching from class I to II, i.e. AH8.1(A*01-C*07-B*08-DRB3*01:01-DRB1*03:01-DQA1*05:01-DQB1*02:01) and AH7.1(C*07-B*07-DRB1*15:01-DQA1*01:02-DQB1*06:02) [14]–[16]. The class II alleles on these haplotypes were excluded in the final model in the unconditional regression analyses, suggesting that the class I alleles, i.e. HLA-B*08 and HLA-B*07, most likely represent the causal variants on these haplotypes. The unconditional logistic regression analyses also revealed a novel class I association with the HLA-C*06 allele. Interestingly, an association with HLA-C*06 is also seen in psoriasis [35], replication of this finding is however necessary.

Supporting evidence for HLA-B*08, or a variant at a nearby locus, being the primary risk variant on AH8.1 in PSC is as follows; (i) the association of HLA-B*08, and not DRB1*03:01, is observed in African American PSC patients [36], a population with generally lower LD levels, and (ii) the localization of the peak association signal in, or in the vicinity, of *HLA-B* in both of the genome-wide association studies that have been conducted in PSC [30], [31], as well as in the recently published PSC Immunochip study [3]. With the exception of DRB3*01:01, HLA-B*08 was also the most significantly associated allele of the genotyped alleles on the AH8.1 in the primary association analysis of the conditional analytic strategy. The DRB3*01:01 allele is also found on the same haplotype as HLA-DRB1*13:01, a known risk variant in PSC [15], [16], and its superior association is attributable to its presence on both the AH8.1 and DRB1*13:01 haplotype. Still, DRB3*01:01 is not likely to be a common risk denominator for these two haplotypes for at least two reasons. Firstly, similar associations have been found for the DRB3*01:01-DRB1*13:01 and DRB3*02:01-DRB1*13:01 haplotypes [16], as also suggested by the lack of LD between DRB3*01:01 and DRB1*13:01 (*r*2 = 0.04 in our controls). Secondly, our analysis showed that there are persistent residual associations for DRB1*13:01 and DQB1*06:03 after conditioning on DRB3*01:01.

A primary role for HLA-B*07 on the AH7.1 is not as evident as for HLA-B*08 on the AH8.1. The class II DRB1*15:01-DQB1*06:02 haplotype showed associations in one of the conditional analysis strategies (strategy III), analogous to additional association signals previously observed with this class II haplotype after removing primary PSC-associated haplotypes from the study populations (i.e. AH8.1 and DRB1*13:01-DQB1*06:03 positive individuals) [16]. Taken together with the strength of the amino acid associations, the possibility remains that for AH8.1 and AH7.1, additional class II effects may exist.

The second most significant association with a HLA haplotype in both analytic approaches was with the DRB1*13:01-DQB1*06:03 haplotype. Although this association was largely confined to class II, as the extent of LD is low on this haplotype, genes other than DRB1 and DQB1 may be causative. Still, the association with the DRB1*13:01 allele was slightly stronger than that for the DRB1*13:01-DQB1*06:03 haplotype and the DQB1*06:03 allele in the conditional analytic strategy. Also, given the association with DRB1*13:01 in African Americans, who have comparable frequencies of DQB1*05:01 and DQB1*06:03 in conjunction with DRB1*13:01 [37], it may be speculated that *DRB1* is the causative locus on this class II haplotype.

The most consistent protective association with PSC has been with the DRB1*04-DQB1*03 haplotype [14]–[16]. This was also evident in the current study where the DRB1*04-DQB1*03 haplotype was associated in both the unconditional and conditional analytic approach. DRB1*04:01, DQB1*03:01 and DQB1*03:02 all obtained the study-specific significance threshold in the primary association analysis of the conditional analytic strategy, with DRB1*04:04 being borderline significant. The most significant and consistent protective association was with the DQB1*03:01 allele, which could be explained by its frequent co-occurrence with both DRB1*04:01 and DRB1*11:01 in Caucasians. A protective association with DRB1*11 has previously been reported in PSC [17]. However, in the present analysis, additional protective effects, besides the DRB1*04-DQB1*03 haplotype, could only be ascribed to the previously reported HLA-DRB1*07:01-DQA1*02:01-DQB1*03:03 haplotype [15]. The protective effects of the DRB1*04 and DRB1*07 associated haplotypes in the unconditional analyses are particularly notable, since the regression model intrinsically corrects for frequency biases due to high frequencies of other alleles (displacement effect). Hence, the negative associations with these two haplotypes are likely to represent biological effects that are truly protective against PSC.

This study does not attempt to map disease predisposition to particular amino acid variants in proteins encoded by PSC-associated HLA alleles. Rather, the present study incorporated previously implicated amino acid variants (position 37 and 86) in DRβ [18], and aimed to explore the relationship between these and the full MHC architecture in PSC by also incorporating other previously published candidate loci/variants (e.g. the MICA5.1 allele). Asparagine, Tyrosine and Serine at position 37 were all significantly associated in several conditional strategies, in addition to Valine and Glycine at position 86, in accordance with previous results [18]. In the third conditional strategy, two amino acids, Asparagine and Serine at position 37, accounted for most of the association signal. Still, attributing most of the MHC association in PSC to these two amino acids would be incorrect. First, we have not directly compared this contribution to that of amino acid variants at HLA loci other than *DRB1*. Additionally, both the present and previous analyses provide considerable evidence of a strong class I contribution to the AH8.1 association in PSC, meaning that Asparagine at position 37 is unlikely to represent the full explanation. Still, the significance of the amino acid variants at position 37 and 86 seems highly robust and could be an important explanation for the class II associations in PSC.

Increases in study size and SNP density alone have only to some extent proven beneficial in the detailed resolving of MHC associations [3]. The lack of a conclusive resolution along with the strength of the statistical associations suggests, that rather than a lack of statistical power *per se*, there is a need for yet more refined analytical strategies. We therefore chose to explore the utility of various statistical approaches in a population of uniform geographical origin and for whom SNP mapping data as well as direct, sequencing-based HLA and MICA microsatellite typing were available. Unconditional, multi-locus regressions of HLA alleles have not previously been performed and did show the unquestionable presence of independent class I and class II associations. Similar approaches are likely to be useful in other MHC associated conditions for which a primary association has not been established. Several different conditional regressions were presented in full to highlight the impact of analytical strategy on the results, illustrating that one should be cautious when concluding on “causal” loci within the MHC based on statistical evidence alone. The discourse facilitated by the three strategies as a whole nevertheless allowed for a relatively robust detection of the novel HLA class III PSC risk locus as well as corroboration of findings from the unconditional multivariate analysis. The most important limitation to our study, as also apparent in all studies based on HLA imputation from SNP data (see S3 Table), is that only a subset of the class II genes has been fully typed. Complete HLA class II sequencing in study populations of multiple ethnicities [38] is needed to establish which HLA class II determinants may be primarily involved in PSC. Technology is improving [39], and a major challenge for the International PSC study group (www.ipscsg.org) moving forward will be to expand its DNA collection to involve non-Caucasian and admixed patient populations.

## Conclusion

By application of multiple statistical strategies, we were able to comprehensively characterize the genetic architecture of the MHC-associated susceptibility to PSC. A new finding is the likely existence of a distinct HLA class III risk locus in the vicinity of *NOTCH4*. Further, by using unconditional models in assessing the MHC associated susceptibility, new information was obtained concerning which HLA class I and II loci may be primarily involved. The identification of the primary risk alleles at the *HLA-B* and -*DRB1* loci should facilitate further work to characterize which antigens may be causing the immune responses involved in PSC development.

## Supporting Information

S1 Fig **Association analyses within the major histocompatibility complex (MHC) in primary sclerosing cholangitis (PSC) after conditioning on rs116212904.** The conditional *P*-values (y-axis) are plotted against the position on chromosome 6 shown in million base pairs (Mb, x-axis). HLA alleles, *DRB1-DQB1* haplotypes, SNPs, the MICA5.1 allele and killer immunoglobulin-like receptor ligand properties for HLA-B and HLA-C are designated by distinct symbols and colors as shown in legend. The Bonferroni-corrected statistical significance threshold (*P*≤2.66×10^−6^) is indicated by the black, horizontal dotted line.
(TIF)

S1 Table Genotype distributions for the negatively associated DRB1*04, DRB1*11 and DRB1*07 alleles in patients with primary sclerosing cholangitis (PSC) as compared to healthy controls.
(DOCX)

S2 Table *HLA-A, HLA-B, HLA-C, HLA-DRB1, HLA-DRB3 and HLA-DQB1* allele frequencies in patients with primary sclerosing cholangitis (n = 365) and healthy controls (n = 368).
(DOCX)

S3 Table Coverage of HLA class II genes (designated by X) by current study and two commonly employed single nucleotide polymorphism (SNP) based imputation algorithms; HLA*IMP2 and SNP2HLA.
(DOCX)
