# Supplementary material for: Refinement of the MHC Risk Map in a Scandinavian Primary Sclerosing Cholangitis Population
Source: PLoS One. 2014 Dec 18;9(12):e114486. doi: 10.1371/journal.pone.0114486 (PMC4270690; doi:10.1371/journal.pone.0114486)
Supplement: S1 Table — Genotype distributions for the negatively associated DRB1*04, DRB1*11 and DRB1*07 alleles in patients with primary sclerosing cholangitis (PSC) as compared to healthy controls. (DOCX) [file pone.0114486.s002.docx]

**Table S1.** Genotype distributions for the negatively associated DRB1*04, DRB1*11 and DRB1*07 alleles in patients with primary sclerosing cholangitis (PSC) as compared to healthy controls.

| *DRB1* allele 1 | *DRB1* allele 2 | PSC  n alleles | Healthy controls  n alleles | Odds ratio  (95% CI)^*^ | Uncorrected  *P* value^*^ |
| --- | --- | --- | --- | --- | --- |
| 04 | 13:01 | 16 | 9 | 1.8 (0.8, 4.0) | 0.15 |
| 04 | 03:01 | 16 | 17 | 0.9 (0.5, 1.9) | 0.88 |
| 04 | 15:01 | 7 | 25 | 0.3 (0.1, 0.6) | 0.002 |
| 04 | X^†^ | 13 | 108 | 0.1 (0.05, 0.2) | 10^-15^ |
| 07 | 13:01 | 3 | 0 | 7.1 (0.8, 64) | 0.08 |
| 07 | 03:01 | 6 | 8 | 0.8 (0.3, 2.1) | 0.60 |
| 07 | 15:01 | 3 | 5 | 0.6 (0.2, 2.3) | 0.49 |
| 07 | X^†^ | 4 | 30 | 0.1 (0.05, 0.4) | 0.00005 |
| 11 | 13:01 | 8 | 2 | 3.5 (0.9, 13) | 0.06 |
| 11 | 03:01 | 7 | 8 | 0.9 (0.3, 2.3) | 0.81 |
| 11 | 15:01 | 6 | 11 | 0.6 (0.2, 1.5) | 0.23 |
| 11 | X^†^ | 7 | 31 | 0.2 (0.1, 0.5) | 0.0002 |

*Odds ratio and *P* values were calculated based on the total data set comparing patients versus healthy control genotypes.

^†^ X represents non-DRB1*1301, non-DRB1*0301 and non-DRB1*1501 alleles.
