# Supplementary material for: Refinement of the MHC Risk Map in a Scandinavian Primary Sclerosing Cholangitis Population
Source: PLoS One. 2014 Dec 18;9(12):e114486. doi: 10.1371/journal.pone.0114486 (PMC4270690; doi:10.1371/journal.pone.0114486)
Supplement: S2 Table — HLA-A, HLA-B, HLA-C, HLA-DRB1, HLA-DRB3 and HLA-DQB1 allele frequencies in patients with primary sclerosing cholangitis (n = 365) and healthy controls (n = 368). (DOCX) [file pone.0114486.s003.docx]

**Table S2a.** *HLA-A* allele frequencies in patients with primary sclerosing cholangitis (n=365) and healthy controls (n=368).

| *HLA-A** | PSC  n alleles (%) | Healthy controls  n alleles (%) | Odds ratio (95% CI) |
| --- | --- | --- | --- |
| 1 | 218 (29.9) | 119 (16.2) | 2.3 (1.7, 2.9) |
| 2 | 200 (27.4) | 241 (32.7) | 0.79 (0.6, 1.0) |
| 3 | 100 (13.7) | 116 (15.8) | 0.86 (0.6, 1.2) |
| 9 | 60 (8.2) | 78 (10.6) | 0.77 (0.5, 1.1) |
| 10 | 25 (3.4) | 34 (4.6) | 0.75 (0.4, 1.3) |
| 11 | 34 (4.7) | 41 (5.6) | 0.84 (0.5, 1.3) |
| 19 | 55 (7.5) | 71 (9.6) | 0.78 (0.5, 1.1) |
| 28 | 28 (3.8) | 35 (4.8) | 0.81 (0.5, 1.3) |

*HLA-A presented with serotype nomenclature, i.e. A1 corresponds to HLA-A*01:XX
alleles, A2 to A*02:XX, A3 to A*03:XX, A9 to A*23:XX and A*24:XX, A10 to A*25:XX, A*26:XX, A*34:XX and A*66:XX, A11 to A*11:XX, A19 to A*29:XX, A*30:XX, A*31:XX, A*32:XX, A*33:XX and A*74:XX, A28 to A*68:XX.

CI: confidence interval, PSC: primary sclerosing cholangitis.

**Table S2b.** *HLA-B* allele frequencies in patients with primary sclerosing cholangitis (n=365) and healthy controls (n=368).

| *HLA-B** | PSC  n alleles (%) | Healthy controls  n alleles (%) | Odds ratio (95% CI) |
| --- | --- | --- | --- |
| 5 | 25 (3.5) | 26 (3.6) | 1.0 (0.6, 1.7) |
| 7 | 116 (16.4) | 118 (16.1) | 1.0 (0.7, 1.3) |
| 8 | 243 (34.3) | 94 (12.9) | 3.4 (2.6, 4.4) |
| 12 | 58 (8.2) | 110 (15.0) | 0.5 (0.4, 0.7) |
| 13 | 6 (0.8) | 4 (0.5) | 1.5 (0.5, 4.6) |
| 14 | 10 (1.4) | 21 (2.9) | 0.5 (0.2, 1.0) |
| 15 | 62 (8.7) | 77 (10.5) | 0.8 (0.6, 1.1) |
| 16 | 8 (1.1) | 17 (2.3) | 0.5 (0.2, 1.1) |
| 17 | 16 (2.3) | 21 (2.9) | 0.8 (0.4, 1.5) |
| 18 | 19 (2.7) | 17 (2.3) | 1.1 (0.6, 2.1) |
| 21 | 6 (0.8) | 15 (2.1) | 0.4 (0.2, 1.0) |
| 27 | 29 (4.1) | 58 (7.9) | 0.5 (0.3, 0.77) |
| 35 | 35 (4.9) | 56 (7.7) | 0.6 (0.40, 1.0) |
| 37 | 15 (2.1) | 12 (1.6) | 1.3 (0.6, 2.6) |
| 40 | 47 (6.6) | 75 (10.3) | 0.6 (0.4, 0.9) |
| 41 | 1 (0.1) | 2 (0.3) | 0.6 (0.1, 3.6) |
| 47 | 0 (0) | 1 (0.1) | 0.3 (0.03, 3.7) |
| 48 | 0 (0) | 2 (0.3) | 0.2 (0.02, 1.9) |
| 53 | 1 (0.1) | 0 (0) | 3.0 (0.3, 33.5) |
| 55 | 7 (1.0) | 2 (0.3) | 3.0 (0.8, 11.5) |
| 56 | 5 (0.7) | 2 (0.3) | 2.2 (0.6, 8.9) |

*HLA-B presented with serotype nomenclature, i.e. B5 corresponds to HLA-B*51:XX and B*52:XX alleles, B7 to B*07:XX, B8 to B*08:XX, B12 to B*44:XX and B*45:XX, B13 to B*13:XX, B14 to B*14:XX, B15 to B*15:XX, B16 to B*38:XX and B*39:XX, B17 to B*57:XX and B*58:XX, B18 to B*18:XX, B21 to B*49:XX and B*50:XX, B27 to B*27:XX, B35 to B*35:XX, B37 to B*37:XX, B40 to B*40:XX, B41 to B*41:XX, B47 to B*47:XX, B48 to B*48:XX, B53 to B*53:XX, B55 to B*55:XX, B56 to B*56:XX.

CI: confidence interval, PSC: primary sclerosing cholangitis.

**Table S2c.** *HLA-C* allele frequencies in patients with primary sclerosing cholangitis (n=365) and healthy controls (n=368).

| *HLA-C* | PSC  n alleles (%) | Healthy controls  n alleles (%) | Odds ratio (95% CI) |
| --- | --- | --- | --- |
| 01 | 28 (3.8) | 28 (3.8) | 1.0 (0.6, 1.7) |
| 02 | 28 (3.8) | 45 (6.1) | 0.6 (0.4, 1.0) |
| 03 | 107 (14.7) | 130 (17.6) | 0.8 (0.6, 1.1) |
| 04 | 47 (6.4) | 73 (9.9) | 0.6 (0.4, 0.9) |
| 05 | 28 (3.8) | 56 (7.6) | 0.5 (0.3, 0.8) |
| 06 | 47 (6.4) | 39 (5.3) | 1.2 (0.8, 1.9) |
| 07 | 365 (50.0) | 246 (33.3) | 2.0 (1.6, 2.5) |
| 08 | 14 (1.9) | 37 (5.0) | 0.4 (0.2, 0.7) |
| 12 | 15 (2.1) | 21 (2.8) | 0.7 (0.4, 1.4) |
| 14 | 9 (1.2) | 4 (0.5) | 2.1 (0.7, 6.3) |
| 15 | 13 (1.8) | 13 (1.8) | 1.0 (0.5, 2.1) |
| 16 | 8 (1.1) | 13 (1.8) | 0.6 (0.3, 1.5) |
| 17 | 1 (0.1) | 1 (0.1) | 1.0 (0.1, 7.1) |

CI: confidence interval, PSC: primary sclerosing cholangitis.

**Table S2d.** *HLA-DRB1* allele frequencies in patients with primary sclerosing cholangitis (n=365) and healthy controls (n=368).

| *HLA-DRB1* | PSC  n alleles (%) | Healthy controls  n alleles (%) | Odds ratio (95% CI) |
| --- | --- | --- | --- |
| 01:01 | 59 (8.1) | 77 (10.5) | 0.75 (0.53, 1.1) |
| 01:02 | 3 (0.4) | 7 (1.0) | 0.47 (0.14, 1.6) |
| 01:03 | 9 (1.2) | 10 (1.4) | 0.91 (0.38, 2.2) |
| 01:07 | 0 (0) | 1 (0.1) | 0.34 (0.03, 3.7) |
| 03:01 | 255 (34.9) | 106 (14.4) | 3.2 (2.5, 4.1) |
| 04:01 | 36 (4.9) | 93 (12.6) | 0.36 (0.24, 0.54) |
| 04:02 | 1 (0.1) | 1 (0.1) | 1.0 (0.14, 7.2) |
| 04:03 | 3 (0.4) | 6 (0.8) | 0.54 (0.16, 1.9) |
| 04:04 | 8 (1.1) | 47 (6.4) | 0.17 (0.08, 0.35) |
| 04:05 | 0 (0) | 3 (0.4) | 0.14 (0.01, 1.3) |
| 04:07 | 2 (0.3) | 3 (0.4) | 0.72 (0.16, 3.2) |
| 04:08 | 1 (0.1) | 5 (0.7) | 0.27 (0.05, 1.4) |
| 07:01 | 29 (4.0) | 55 (7.5) | 0.52 (0.33, 0.82) |
| 08:01 | 10 (1.4) | 29 (3.9) | 0.35 (0.17, 0.70) |
| 08:02 | 1 (0.1) | 1 (0.1) | 1.0 (0.14, 7.2) |
| 08:03 | 1 (0.1) | 0 (0) | 3.0 (0.3, 33.5) |
| 08:04 | 0 (0) | 2 (0.3) | 0.20 (0.02, 1.9) |
| 09:01 | 12 (1.6) | 6 (0.8) | 2.0 (0.78, 4.9) |
| 10:01 | 4 (0.5) | 5 (0.7) | 0.82 (0.25, 2.7) |
| 11:01 | 10 (1.4) | 32 (4.3) | 0.32 (0.16, 0.63) |
| 11:02 | 1 (0.1) | 3 (0.4) | 0.43 (0.08, 2.4) |
| 11:03 | 1 (0.1) | 4 (0.5) | 0.33 (0.06, 1.7) |
| 11:04 | 5 (0.7) | 4 (0.5) | 1.2 (0.38, 4.1) |
| 12:01 | 5 (0.7) | 16 (2.2) | 0.33 (0.13, 0.84) |
| 13:01 | 119 (16.3) | 47 (6.4) | 2.8 (2.0, 4.0) |
| 13:02 | 16 (2.2) | 39 (5.3) | 0.41 (0.23, 0.73) |
| 13:03 | 2 (0.3) | 5 (0.7) | 0.46 (0.11, 1.8) |
| 14:01 | 9 (1.2) | 12 (1.6) | 0.76 (0.33, 1.8) |
| 14:02 | 0 (0) | 1 (0.1) | 0.34 (0.03, 3.7) |
| 15:01 | 124 (17.0) | 109 (14.8) | 1.2 (0.89, 1.6) |
| 15:02 | 0 (0) | 3 (0.4) | 0.14 (0.02, 1.3) |
| 16:01 | 2 (0,3) | 1 (0.1) | 1.7 (0.28, 10.1) |

CI: confidence interval, PSC: primary sclerosing cholangitis.

**Table S2e.** *HLA-DRB3* allele frequencies in patients with primary sclerosing cholangitis (n=365) and healthy controls (n=368).

| *HLA-DRB3* | PSC  n alleles (%) | Healthy controls  n alleles (%) | Odds ratio (95% CI) |
| --- | --- | --- | --- |
| 01:01 | 307 (0.42) | 128 (0.17) | 3.4 (2.7, 4.4) |
| 02:01 | 3 (0.004) | 4 (0.005) | 0.8 (0.2, 2.9) |
| 02:02 | 93 (12.7) | 91 (0.12) | 1.0 (0.8, 1.4) |
| 03:01 | 15 (0.02) | 41 (0.06) | 0.4 (0.2, 0.6) |
| Null | 307 (0.42) | 470 (0.64) | 0.4 (0.3, 0.5) |

CI: confidence interval, PSC: primary sclerosing cholangitis.

**Table S2f.** *HLA-DQB1* allele frequencies in patients with primary sclerosing cholangitis (n=365) and healthy controls (n=368).

| *HLA-DQB1* | PSC  n alleles (%) | Healthy controls  n alleles (%) | Odds ratio (95% CI) |
| --- | --- | --- | --- |
| 02 | 274 (38.8) | 143 (20.0) | 2.5 (2.0, 3.2) |
| 03:01 | 32 (4.5) | 112 (15.7) | 0.3 (0.2, 0.4) |
| 03:02 | 40 (5.7) | 97 (13.6) | 0.4 (0.3, 0.6) |
| 03:03 | 16 (2.3) | 26 (3.6) | 0.6 (0.3, 1.1) |
| 03:04 | 0 (0) | 1 (0.1) | 0.3 (0.03, 3.7) |
| 03:05 | 0 (0) | 1 (0.1) | 0.3 (0.03, 3.7) |
| 04 | 11 (1.6) | 33 (4.6) | 0.3 (0.2, 0.7) |
| 05:01 | 75 (10.6) | 97 (13.6) | 0.8 (0.6, 1.0) |
| 05:02 | 4 (0.6) | 1 (0.1) | 3.1 (0.6, 15.8) |
| 05:03 | 8 (1.1) | 12 (1.7) | 0.7 (0.3, 1.6) |
| 05:04 | 0 (0) | 1 (0.1) | 0.3 (0.03, 3.7) |
| 06:01 | 1 (0.1) | 1 (0.1) | 1.0 (0.1, 7.2) |
| 06:02 | 115 (16.3) | 106 (14.8) | 1.1 (0.8, 1.5) |
| 06:03 | 112 (15.9) | 47 (6.6) | 2.7 (1.9, 3.8) |
| 06:04 | 17 (2.4) | 35 (4.9) | 0.5 (0.3, 0.9) |
| 06:05 | 0 (0) | 1 (0.1) | 0.3 (0.03, 3.7) |
| 06:09 | 1 (0.1) | 1 (0.1) | 1.0 (0.1, 7.2) |

CI: confidence interval, PSC: primary sclerosing cholangitis.
