# Supplementary material for: Refinement of the MHC Risk Map in a Scandinavian Primary Sclerosing Cholangitis Population
Source: PLoS One. 2014 Dec 18;9(12):e114486. doi: 10.1371/journal.pone.0114486 (PMC4270690; doi:10.1371/journal.pone.0114486)
Supplement: S3 Table — Coverage of HLA class II genes (designated by X) by current study and two commonly employed single nucleotide polymorphism (SNP) based imputation algorithms; HLA*IMP2 and SNP2HLA. (DOCX) [file pone.0114486.s004.docx]

**Table S3.** Coverage of HLA class II genes (designated by X) by current study and two commonly employed single nucleotide polymorphism (SNP) based imputation algorithms; HLA*IMP2 and SNP2HLA.

| Named genes within the HLA class II region | This study | HLA*IMP2 | SNP2HLA |
| --- | --- | --- | --- |
| *DRA* |  |  |  |
| *DRB1* | X | X | X |
| *DRB2* |  |  |  |
| *DRB3* | X | X |  |
| *DRB4* |  | X |  |
| *DRB5* |  | X |  |
| *DRB6** |  |  |  |
| *DRB7** |  |  |  |
| *DRB8** |  |  |  |
| *DRB9** |  |  |  |
| *DQA1* |  | X | X |
| *DQB1* | X | X | X |
| *DQA2* |  |  |  |
| *DQB2* |  |  |  |
| *DQB3* |  |  |  |
| *DOA* |  |  |  |
| *DOB* |  |  |  |
| *DMA* |  |  |  |
| *DMB* |  |  |  |
| *DPA1* |  |  | X |
| *DPB1* |  | X | X |
| *DPA2* |  |  |  |
| *DPB2* |  |  |  |
| *DPA3* |  |  |  |
| *TAP1* |  |  |  |
| *TAP2* |  |  |  |
| *PSMB9* |  |  |  |
| *PSMB9* |  |  |  |

*Pseudogenes.
